# Spotlight on mass spectrometric non‐target screening analysis: Advanced data processing methods recently communicated for extracting, prioritizing and quantifying features

**DOI:** 10.1002/ansa.202200001

**Published:** 2022-04-05

**Authors:** Susanne Minkus, Stefan Bieber, Thomas Letzel

**Affiliations:** ^1^ AFIN‐TS GmbH Augsburg Germany; ^2^ Technical University of Munich (Chair of Urban Water Systems Engineering) Munich Germany

**Keywords:** environmental analysis, feature extraction, non‐target screening, prioritization, semi‐quantification

## Abstract

Non‐target screening of trace organic compounds complements routine monitoring of water bodies. So‐called features need to be extracted from the raw data that preferably represent a chemical compound. Relevant features need to be prioritized and further be interpreted, for instance by identifying them. Finally, quantitative data is required to assess the risks of a detected compound. This review presents recent and noteworthy contributions to the processing of non‐target screening (NTS) data, prioritization of features as well as (semi‐) quantitative methods that do not require analytical standards. The focus lies on environmental water samples measured by liquid chromatography, electrospray ionization and high‐resolution mass spectrometry. Examples for fully‐integrated data processing workflows are given with options for parameter optimization and choosing between different feature extraction algorithms to increase feature coverage. The regions of interest‐multivariate curve resolution method is reviewed which combines a data compression alternative with chemometric feature extraction. Furthermore, prioritization strategies based on a confined chemical space for annotation, guidance by targeted analysis and signal intensity are presented. Exploiting the retention time (RT) as diagnostic evidence for NTS investigations is highlighted by discussing RT indexing and prediction using quantitative structure‐retention relationship models. Finally, a seminal technology for quantitative NTS is discussed without the need for analytical standards based on predicting ionization efficiencies.

## 1 INTRODUCTION

For several decades, the presence of anthropogenic organic chemicals in natural waters is reported. Many of them are known to cause adverse effects on aquatic and maybe even human health.^1^, ^2^ Important prerequisites for mitigating potential risks posed by these chemicals, are comprehensive analysis and monitoring efforts. Known constituents are routinely monitored in waterbodies by (quantitative) target screening methods, mainly based on liquid chromatography (LC), coupled to tandem mass spectrometry (MS/MS), such as triple quadrupole MS. This technique is well established but can cover only a small fraction of the chemical constituents, namely those, which are already known and searched for.^3^ In recent years, LC coupled with soft ionization techniques like electrospray ionization (ESI) hyphenated to high‐resolution MS (HMRS), has become more and more important for the analysis of anthropogenic compounds in the aquatic environment. HRMS instruments are able to accurately screen the entire mass range of small molecules with cycling times short enough to resolve even narrow chromatographic peaks. These technical advancements powered the evolution of non‐target screening (NTS) methods for interrogating samples without any pre‐selected substances.^4^, ^5^ NTS aims at filling the gaps or complementing target screening efforts by allowing to detect so far unexpected or unknown chemical compounds in water bodies as so‐called features. A feature is usually described by the three coordinates accurate mass, retention time (RT) as well as signal intensity. Full scan HRMS measurements produce vast amounts of complex data that need rigorous and sophisticated processing and well‐informed interpretation. The challenge for data evaluation is here to extract, align, filter and bin features that correspond to a real compound and distinguish them from artefacts or redundant signals. Therefore, tools are required to effectively extract, prioritize, annotate and interpret features. Nowadays, there are many examples of different software tools for NTS data evaluation, which help to set up such data processing. On the one hand, there is commercially available software, often marketed by MS vendors, such as Compound Discoverer (Thermo Fisher Scientific), Mass Profiler (Agilent), UNIFI (Waters) or others. On the other hand, there is a rapidly increasing variety of powerful open‐source and/or open‐access software (i.e. XCMS^6^ or MZmine 2^7^), which can be used independently from the applied MS. In the following, the spotlight focuses on advances in such vendor‐independent tools.

Typically, a core NTS data processing strategy includes data pre‐treatment, feature extraction, componentization, feature alignment and filtering. To use data in vendor‐independent tools, data pre‐treatment often entails converting raw HRMS data files into open formats like mzML,^8^ mzXML^9^ or ANDI/netDCF (https://www.astm.org/). Additionally, the data can here be trimmed and simplified through an initial noise threshold or centroiding (i.e. by using the MSconvert tool of the ProteoWizard toolkit^10^). Feature extraction or peak picking often involves extracting ion chromatograms, followed by deconvoluting or integrating a chromatographic peak. There are algorithms taking on that task such as “centWave^11^” implemented in the XCMS tool^6^ or the “ADAP” workflow^12^ within the MZmine 2 framework.^7^ Componentization refers to the binning of redundant ion traces that belong to the same compound such as isotopologues, adducts or in‐source fragments. Features may be aligned across several technical replicates or samples of a batch if they are expected to be the same compound. Finally, filtering feature lists based on various quality criteria (e.g., present in all replicates, absent in the blank samples, minimum signal intensity, etc.) is intended to remove artefacts and false‐positive features. Questions associated with NTS are diverse and require individual data processing solutions. Thus, the processing steps should be handled interchangeable and flexible.

After a feature list of sufficient quality^13^, ^14^ is generated, an NTS method could follow different objectives: Comparative analysis for process evaluation (a), trend analysis (b), or identification of unknown or unexpected substances (c). While objectives a, and b, mainly focus on the comparison of samples and might result in a statistical interpretation, objective c, aims to reveal the identity of specific features and annotate it.

The basis of feature annotation is querying chemical compound databases such as PubChem^15^ (https://pubchem.ncbi.nlm.nih.gov/) or ChemSpider^16^ (https://www.chemspider.com/) by accurate mass. When running MS2 experiments, another dimension of structural information is available in the form of characteristic fragmentation patterns. Spectral libraries like European MassBank^17^ or MassBank of North America (MoNA; https://mona.fiehnlab.ucdavis.edu/) allow a comparison of MS2 spectra. If there are no experimental spectra available, in silico fragmentation prediction performed by i.e. MetFrag^18^ or SIRIUS4 CSI:FingerID,^19^ support tentative identification of features. How confidently a feature could be identified depends on the amount of evidence available and is usually communicated via a level system proposed by Schymanski et al.^20^ A feature is considered unequivocally identified if the proposed structure is confirmed by matching MS1, MS2 and RT information with a reference compound. However, this step constitutes the bottleneck of the workflow as it is time‐consuming and authentic standards can be expensive or even unavailable. To that end, it is necessary to prioritize features that are expected to be environmentally relevant. Criteria for that might be toxicity (T), persistence (P), mobility (M), all combined as PMT, bioaccumulation, endocrine disruption capabilities or the formation of new compounds in processes of biosystems.

To further support the interpretation of LC‐MS data, sophisticated models are increasingly developed and applied. These models are able to mine information from large empirical data sets and describe complex relationships between a chemical structure and biological activity or chromatographic retention. Usually, the data set is split into multiple subsets, one called the training set for fitting the model and another one called the test set which is withheld for external validation of the predictive ability of the model. The training set itself is often submitted to resampling methods such as cross‐validation or bootstrapping for internal validation and definition of the applicability domain.^21^, ^22^

An assortment of recent and – according to the authors' assessment – noteworthy advancements is collected, related to feature extraction (Section 1), prioritization (Section 2) and quantification (Section 3) in NTS analysis. In Section 1, centroiding, as a data pretreatment step comes to fore and software platforms, are presented that combine various feature extraction tools for more effective processing of environmental NTS data. Furthermore, a multivariate chemometric method is discussed as an effective alternative for processing complex LC‐MS data. In Section 2, suggestions are given on how to confine the chemical space in order to streamline the feature identification workflow. When dealing with feature prioritization, a focus lies on methods that take chromatographic information into account. From intensity‐based prioritization, this review moves to a quantification method suited for NTS applications as it refrains from using analytical standards Section 3.

## 2 FEATURE EXTRACTION

If not performed during data acquisition, centroiding is an integral part of LC‐MS data pretreatment. Data size and density are reduced by converting the mass profile peak into a single data point described by mass and intensity. To calculate centroid values, an appropriate peak shape model and the respective parameter settings need to be selected which could become even more complex if peaks are noisy, skewed or overlapping.^23^ Open source centroiding approaches are often considered inferior to vendor approaches as some proprietary information might not be translated to open formats and information on mass peak widths is lacking.^24^, ^25^ The open‐source tool Cent2Prof was developed to convert profile and centroided data in both directions.^25^ Furthermore, a random forest regression model uses mass‐to‐charge (*m/z*) values, relative intensities and retention factors to predict mass peak widths, which could be helpful information further down the feature extraction workflow. The model was internally validated via 500 bootstrapping samples and 5‐fold cross‐validation and the external validation yielded an average prediction error of 56%. However, the model is currently developed based on data acquired with quadrupole time‐of‐flight mass analyzers and needs to be extended for Orbitrap instruments.

**FIGURE 1:**
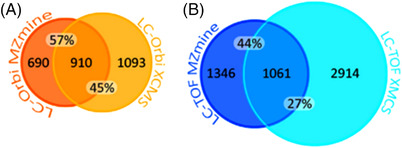
Venn diagrams of the total number of features (areas of the circles) extracted with MZmine 2 and XCMS from data acquired on an LC‐Orbitrap (A) setup and a liquid chromatography–time‐of‐flight (LC‐TOF) setup (B). Reprinted and adapted with permission from Elsevier^27^

As nowadays numerous software tools are available, many of them running on different (sometimes proprietary) algorithms, different results might be expected. Hohrenk et al. compared the four different processing tools enviMass, MZmine 2, XCMS online and the commercial software Compound Discoverer (Thermo Fisher Scientific).^26^ They found that overlapping features constitute only 8%–12% of all extracted features. Even when the 100 most intense features were considered, the percentage of overlapping features did not significantly increase. In the course of finding biomarkers in bioreactor samples, Neusüß and coworkers compared MZmine 2 and XCMS online on two different instrumental setups: LC‐Orbitrap and LC‐TOF.^27^ They found 45%–57% of commonly extracted features for LC‐Orbitrap and 27%–44% for LC‐TOF (see Figure 1). However, the overlap was significantly increased for features prioritized by a multivariate chemometric method. Therefore, partial least square regression was applied to build models for the prediction of gas yields based on feature intensity. Features were then ranked by their importance based on the variable importance in projection which describes a feature's contribution to the model. The small fraction of commonly extracted features underline the need for introducing quality control (QC) measures to an NTS data processing workflow. In both studies, parameters and filters for feature extraction were set to the same values (if possible) for better comparison. Software‐specific parameters were optimized based on the recovery of internal standards. Hohrenk et al. additionally considered verified suspects as well as the total number of features. Those are agreed upon QC criteria that aim at assessing and minimizing false negative and false positive rates, respectively.^14^, ^28^ Individually optimized parameter settings cannot be excluded as a source of variation between feature lists and Hohrenk et al. furthermore suspected varying effects of the blank and replicate filters as they are implemented differently in the investigated software tools. Moreover, the typical QC regime for NTS investigations of environmental samples currently lacks criteria to evaluate the componentization step. Nevertheless, there is reason to assume that the different operations of peak picking algorithms themselves lower the congruence between extracted feature lists.

**FIGURE 2:**
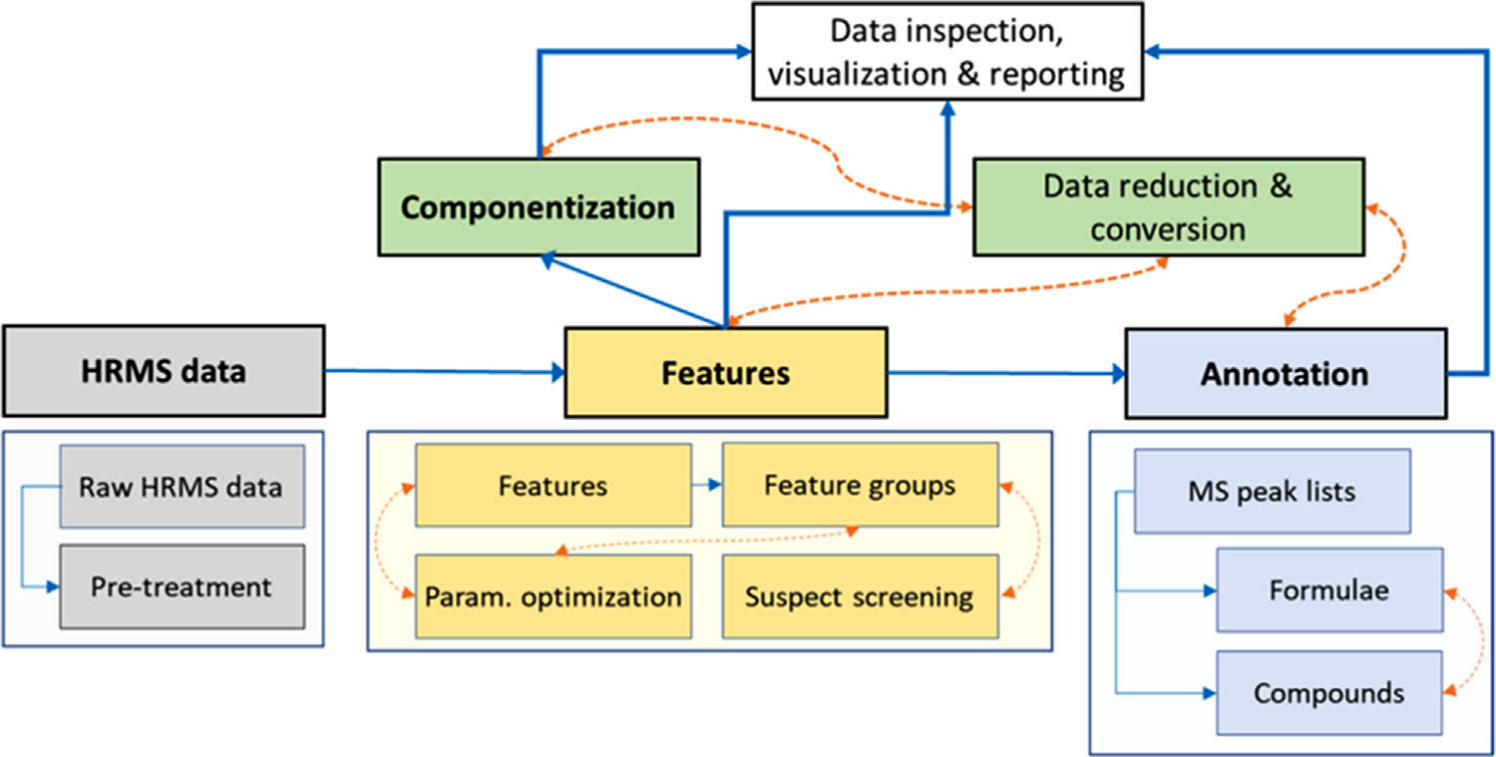
patRoon workflow for processing environmental non‐target screening (NTS) data. Reprinted^30^

The NTS investigator could counter these uncertainties by (a) a thorough QC and reporting routine for data processing (as proposed^14^, ^29^), (b) a deeper understanding of algorithms and (c) orthogonally constructed tools like patRoon.^30^ The open‐source platform patRoon combines multiple established algorithms in an R interface.^31^ As visualized in Figure 2, it covers the entire workflow from data pre‐treatment, feature extraction and componentization, annotation, visualization and reporting. Many open‐source and/or open‐access software tools such as MZmine were originally developed for proteomic or metabolomic research. Consequently, they might lack specific functionalities necessary to meet the demands of environmental analysis such as detection of analytes at low or varying concentrations, trend recognition over longer time periods (i.e. surface water monitoring activities) or across diverse matrices (i.e. influents and effluents of wastewater treatment plants) and annotation of environmentally relevant compounds. Like enviMass,^32^ patRoon was more specifically designed for processing environmental NTS data. By applying strategies for parallelization of processes or threads such as multiprocessing execution times were significantly reduced for several processing steps. For almost all integrated tools, execution times normalized to sequential results were reduced by approximately 200%–500% when increasing the number of parallel processes, with an optimum at the number equal to the number of physical CPU cores (here six). User‐friendliness in patRoon is promoted by partially implementing a graphical user interface (GUI) and an installation script for Microsoft Windows and its dependencies. Furthermore, the results of previous processing steps are accessible at any point throughout the workflow and can be visualized. This is a helpful tool in the development of data evaluation workflows because it can be used to assess the impact of different workflow stages on the results. A noteworthy addition to the patRoon workflow is the adapted R package IPO for automatic optimization of data processing parameters based on the statistical design of experiments (DoE).^33^ A similar functionality was included in another LC‐MS data processing workflow named SLAW, presented by Delabriere et al.^34^ It containerizes several tools to reduce computational time and memory consumption. Apart from the number of detected isotopic peaks as an optimization criterion for peak picking, in SLAW a score was added for the reliability of integration across pooled QC samples, which promotes reproducibility. Objectives, designs or instrumental stability can vary from one NTS study to another which requires specific adjustment of parameter settings. Generally, an integrated optimization method based on DoE and response surface modelling constitutes a more systematic alternative to approaches like “trial and error” or “changing one factor at a time”, which heavily rely on the user's experience, are prone to error (i.e. by not considering statistical interactions) and time‐consuming. In SLAW just like in patRoon, one can choose from different peak picking algorithms. In patRoon, features or annotations derived from different algorithms (i.e. XCMS^6^ or OpenMS^35^) can be compared and a consensus is created. Thereby it is possible to exclusively keep commonly found features or use complementary algorithms to increase feature coverage. This functionality could address the aforementioned concern of little correspondence between different algorithms.

Many feature extraction methods employ a strategy referred to as “binning” to compress LC‐MS data and transform it into a matrix (x = RT; y = *m/z*). Thereby, the *m/z* axis is discretized into bins of a specific size. Yet, finding the appropriate bin size is complicated, chromatographic peaks might be overlaid or split between bins and the approach is prone to spectral inaccuracies.^11^ An alternative strategy for filtering, compressing and resolving LC‐MS data in regions of interest‐multivariate curve resolution (ROIMCR), a method originally introduced to the field of metabolomics.^36^, ^37^ The approach employs two steps: First, data is compressed by searching for “regions of interest” (ROI) which describe areas of densely arranged data points followed by “data voids” as was initially introduced by Stolt et al.^38^ and later on incorporated into the centWave algorithm as part of the XCMS tool.^11^ In the second step, the compressed data is analyzed using multivariate curve resolution‐alternating least squares (MCR‐ALS). With MCR‐ALS, pure spectral and elution profiles of analytes across different chromatographic runs can be recovered, even if they differ in RT and peak shape.^39^ Thus, the MCR‐ALS approach does not require chromatogram deconvolution (shape modelling peaks by i.e. fitting a Gaussian curve or its derivative) and peak alignment across multiple samples. However, MCR methods are not free of uncertainty, for which rotational ambiguity is a major source. It refers to multiple feasible solutions for the bilinear decomposition of data matrices and needs to be reduced by introducing constraints.^40^ Nevertheless, this chemometric approach has been recently picked up for the non‐targeted analysis of proteins^41^ and trace organic compounds in wastewater^42^ due to its ability to overcome coelution problems in complex matrices. An MCR‐ALS tool was implemented in the MATLAB environment with GUI features and is available on http://www.mcrals.info/ (including background information).

## 3 PRIORITIZATION

The enormous amount of information that can be retrieved from NTS data needs to undergo various data reduction steps that should result in a manageable number of relevant features. This process can be defined as prioritization in its broadest sense. A form of prioritization is already introduced into the feature extraction workflow when setting input parameters and applying filters to comply with certain quality standards. For instance, analyzing pooled samples as it is general practice in metabolomics since 2006,^43^, ^44^ has made its way into the QC regime of environmental studies as well.^14^, ^45^, ^46^ Strategies downstream of feature extraction are often based on metadata, physicochemical properties, spatial and temporal trends, suspected occurrence, identification confidence or anticipated effects.

Especially when following an identification workflow, feature prioritization is crucial as compound annotation in the face of the chemical universe can become a daunting task. PubChem as one of the largest chemical inventories currently contains over 100 million compounds which could mean thousands of matches per mass query. Nevertheless, these compound databases are necessary for annotating features since they provide additional information on physicochemical properties and metadata of compounds for filtering or ranking. Narrowing the scope of expected compounds to a sub‐group of major interest or relevance could be the first step in streamlining the identification process. Smaller and specialized compound databases, such as STOFF‐IDENT^47^ or CompTox,^48^ could facilitate identifying environmentally relevant compounds. Recently, Schymanski et al. collapsed the PubChem database into PubChemLite by selecting relevant subcategories such as “Agrochemical Information” or “Drug and Medication Information”.^49^ So far, the PubChemLite can be incorporated into workflows with MetFrag or patRoon. At the risk of missing some correct matches, it might streamline identification workflows by increasing computational efficiency as well as ranking performance.

**FIGURE 3:**
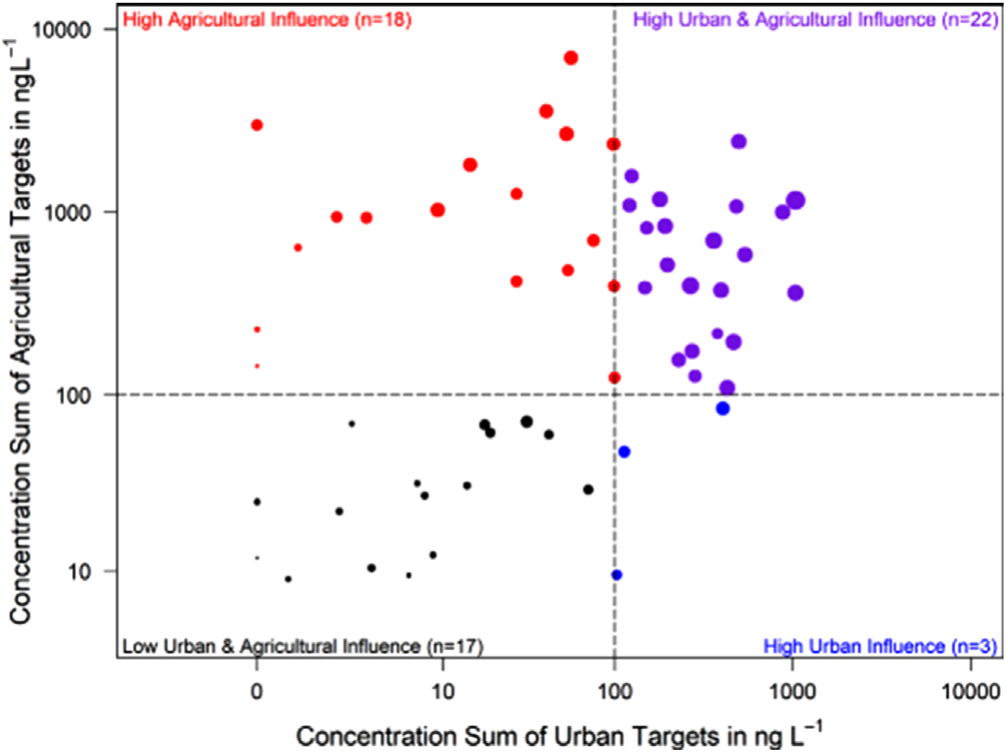
Classification of 60 groundwater monitoring sites based on targets of urban and agricultural origin. The dot size corresponds to the number of targets and is scaled to a maximum of 74. Sum concentrations of 100 ng L^–1^ mark the thresholds (dashed lines) for the four classes. Reprinted with permission from Elsevier^46^

Source‐related prioritization is another approach for limiting the space of expected chemicals prior to structural elucidation. Kiefer et al. classified groundwater samples based on urban or agricultural influence. Therefore, they calculated the sum concentrations for target compounds of urban origin (i.e. sweeteners and industrial compounds) and for targets of agricultural origin (pesticides and their transformation products). If the sum concentration exceeded 100 ng L^–1^ a sample was considered to be highly influenced (see Figure 3). The classification system allowed to prioritize features for further structural elucidation based on suspected urban and/or agricultural origin.^46^

Accurate mass alone is not sufficient for excluding false compound candidates. Even if a mass spectrometer had a theoretical mass accuracy of 0.1 ppm, it would still result in multiple possible elemental compositions for features with masses >500 Da.^50^ Furthermore, a query of the PubChem database via MetFrag for the formula C17H29NO4 (311.4 g mol^–1^) obtained 1304 candidate compounds (accessed on 21 December 2021). The manifold annotation possibilities suggest that additional diagnostic evidence is needed for ranking and filtering non‐target features. Chromatographic parameters such as RT can be connected to the physicochemical properties of an analyte. Our group demonstrated the benefits of this relationship by filtering for polar compound candidates.^51^, ^52^ NTS measurements were carried out on a chromatographic setup comprising reversed‐phase LC (RPLC) coupled in series to hydrophilic interaction liquid chromatography (HILIC) for the separation of analytes of an extended polarity range.^53^ Features eluting from the HILIC column were annotated using a compound database specialized in water analysis, namely the STOFF‐IDENT^47^ on the FOR‐IDENT platform (https://water.for‐ident.org). Only candidates with a negative log *D* value (logarithmic distribution coefficient between water and octanol at pH 7) were further considered. The log *D* filter reduced the number of plausibly annotated features per sample by on average 43%.

Retention time indexing, as originally introduced for gas chromatography by Kováts using *n*‐alkanes,^54^ allows comparing retention behaviour with different laboratories and libraries. The approach has gained traction in the field of LC‐MS as well. By harmonizing the RT and thus making it more comparable, it becomes more accessible to NTS investigations, that is by using it as additional evidence to identify features. Usually, experimental RTs of features are normalized using a linear calibration curve based on a set of standard compounds. An approach implemented in the FOR‐IDENT platform correlates RT indices (RTIs) with log *D* values using experimentally determined calibrants. The observed log *D* values are compared to those of compounds stored in one of the databases hosted by the platform. The difference Δlog *D* can be used to further evaluate the plausibility of compounds assigned to a feature.^55^, ^56^ Aalizadeh et al. introduced an RTI system as well as a prediction approach based on quantitative structure‐retention relationship (QSRR) models.^57^ For each ESI mode, they selected a set of 18 calibrants that represented the chemical space (here 2123 compounds) best based on chemical similarity and maximum overlap with RTs. These calibrants can be used to normalize experimental RTs to RTIs between 1 and 1000. Tests on different RPLC columns, under different chromatographic conditions and in different laboratories found the RTI system harmonize RTs reliably. However, the performance of the RTI systems was less accurate for a mobile phase composed of acetonitrile/H_2_O compared to methanol/H_2_O and the uncertainty was generally increased in ESI negative mode. Two QSRR models were built following previously reported workflows^58^, ^59^ with the objective of predicting RTIs based on one of the most representative molecular descriptors (i.e. Log *D* with the highest relative importance of approximately 68%). The non‐linear model employing a support vector machine (SVM) algorithm outperformed the multiple linear regression algorithm. The external validation for ESI negative and positive mode returned *R* ^2^ values of 0.833 and 0.868 and mean root mean square errors (RMSE) of 75.70 and 82.64, respectively. Comparing experimental RTI between laboratories or in silico predictions could increase the identification confidence in NTS workflows. Caution should be exercised if calibrants deviate considerably from linearity or compounds fall outside the applicability domain of the RTI model.

Julien Parinet compared various sets of molecular descriptors and five different machine learning algorithms in order to find the combination that most accurately and precisely predicts the RTs of pesticides on an RPLC system.^60^ Six sets of molecular descriptors from four influential studies^61^, ^62^, ^63^, ^64^, ^65^ were chosen and a seventh set was created by merging all variables. The molecular descriptors were calculated for roughly 800 pesticides. The model that predicted RTs best, used all 40 molecular descriptors and multi‐layer perceptron regression. However, a less complex model based on SVM regression showed no significant differences in performance. It only used eight molecular descriptors (Log *P*, log *D*, molecular weight, molecule volume, Molar refractivity, polar surface area, H‐Donor and H‐Acceptors) and thus seemed to be the best compromise. The results of the external validation returned an *R* ^2^ of 0.85, an RMSE value of 0.69 min and a 6% percentage error.

The signal intensity is one dimension of a feature often used for prioritization as higher intensities often produce higher quality MS1 peaks and MS2 spectra and are assumed to be to some extent proportional to a compound's concentration.^3^ Many prioritization efforts in non‐targeted analysis serve the purpose of streamlining the workflow towards a (tentative) identification of features. As this approach heavily relies on chemical databases, it discriminates against so far unreported compounds even though they might be environmentally relevant. For assessing general patterns in technical or natural systems, such as evaluating the effectiveness of wastewater treatment or locating a spilling event in a river, following the entire identification regime may not be necessary or even cut important information. That is why intensity‐based prioritization approaches are not only interesting for identification strategies but also for process evaluations or trend analysis. Non‐linear response relationships and matrix effects like signal suppression or enhancement often hamper the comparison of samples with different matrices. Therefore, strategies need to be developed to scale features to equal intensities. One way of normalizing intensities is based on spiking isotopically labelled internal standards (ISTDs) into each sample that serve as chemical surrogates. An exemplary implementation would be to first calculate the median intensity per ISTD across all samples. Secondly, the deviation of each ISTD intensity in an individual sample from the median across all samples is determined. Finally, the median deviation is calculated for each sample which served as a normalization factor. The normalization factor is then applied to correct the signal intensities of non‐target features.^45^, ^66^, ^67^ Schollée et al. used normalized feature intensities to assign trends for the evaluation of different advanced wastewater treatments.^45^ They, therefore, aligned features along with treatment steps and defined relative ranges of high (1), medium (0) and low (‐1) intensities at 100%–60%, 60%–20% and <20% of the maximum intensity, respectively. These “barcode trends” indicated whether a feature was eliminated, formed or persistent. In a former process evaluation study by Bader et al. in 2017, fold changes of features between effluent and influent samples were determined and fold change intervals were defined for elimination, decrease, consistency, increase and formation.^68^ Reymond et al. brought together intensity‐based and target‐guided prioritization approaches in order to find and identify features in wastewater samples that are related to the synthesis of illicit drugs.^69^ Peak areas were normalized using the closest eluting ISTD and then compared to loads of known target drugs using similarity metrics (Pearson's correlation coefficient, Spearman's rank correlation coefficient, the distance correlation and maximum information coefficient). Thereby, 28 features were detected that were closely associated with the target drug patterns. It should be noted that deciding on the size and composition of a sufficient ISTD set for intensity normalization is difficult, as the compounds in the environmental sample are initially unknown to the investigator and a large structural variety can be expected. Moreover, the factors influencing the RT and the ionization efficiency are to some extent different.^70^, ^71^

## 4 QUANTIFICATION OF NON‐TARGET FEATURES

Semi‐quantitative prioritization methods attempt to overcome the qualitative focus of NTS studies by prioritizing features that are expected to be environmentally relevant as they are (a) present at comparatively high concentrations or (b) newly formed or emitted. These assumptions are based on relative interpretations of chemical abundance, derived from normalization over a sample set or calculation of fold changes for binary comparison. Still, risk assessment and regulatory decisions require absolute quantitative estimates.^72^ As an example, there are well‐known stressors such as steroid hormones that already have ecotoxicological effects on aquatic organisms at concentrations in the ng L^–1^ range or even below.^73^ Moreover, analytical standards for newly identified compounds and transformations products might be commercially unavailable and their synthesis resource‐intensive. Thus, quantification methods are desirable as they do not rely on calibration with an authentic standard. This proved to be a difficult task since the signal response in an LC‐ESI‐HRMS setup not only depends on concentration but also the compound, the sample matrix, the mobile phase composition and the instrument. To overcome these issues Liigand et al. proposed an approach for quantifying compounds based on predicted ionization efficiency.^74^ Ion efficiency data was collected in ESI positive and negative mode from >450 compounds and >100 different mobile phase compositions. A model based on random forest regression was developed with the measured ionization efficiency values, structure‐related chemical descriptors (PaDEL descriptors^75^) and empirical eluent descriptors. The external validation of the ESI positive and negative models, returned RMSE values of 3.0 times and 2.3 times for the test sets, respectively. The authors measured the ionization efficiency *IE* of a compound relative to an anchoring compound and the prediction error was defined as follows^74^:

$$Prediction\;error\;=\;max\left\{\begin{array}{c} \frac{predicted\;IE}{measured\;IE}\\ \frac{predicted\;IE}{measured\;IE} \end{array}\right.$$

Interestingly, the models revealed that the number of hydrogen and nitrogen atoms in a molecule as well as viscosity, pH and presence of ammonium ions in the mobile phase strongly influence the ionization efficiency. The authors were able to show that predicted ionization efficiency values are transferrable between different instruments. Specific instrumental parameters are not accounted for in the ionization efficiency predictions. Therefore, the universal values need to be converted by measuring a set of calibration compounds at defined concentration levels under the same conditions as the compound of interest. Besides compensating for instrumental differences, the authors expect this transformation to also account for matrix effects. Furthermore, they were able to show a rather consistent prediction error across different matrices. The instrument‐specific ionization efficiency value as well as the measured signal intensity of the compound of interest can then be used to estimate its concentration. In a recent study by Kruve et al.,^70^ the aforementioned approach for quantification without analytical standards (a) was compared to two others using the response factors of parent compounds to quantify TPs (b) and of the closest eluting ISTD (c).^76^ Therefore, 74 micropollutants were detected in real groundwater samples and quantified with analytical standards for reference. The quantification approach based on ionization efficiency exhibited the highest prediction accuracy and the narrowest error distribution. Its mean prediction error was a factor of 1.8 and all of the data points had an error below a factor of 10. It should be noted that the model is able to predict ionization efficiency for protonated and charged [M]^+^ compounds in ESI positive mode and for deprotonated species in negative mode but not for sodium or ammonium adducts. The ionization efficiency prediction could complement NTS investigations by allowing estimation of the concentration of tentatively identified features. The semi‐quantification approach could even be applied retrospectively to stored NTS data if calibration information (QC samples and ISTDs) is available.^71^ However, if no SMILES code was putatively assigned to a feature, this method does not apply.

## 5 CONCLUSION AND OUTLOOK

The interest in NTS has been constantly increasing in the last few years. This can be demonstrated by over 1200 publications (SCOPUS search for non(‐)target*/untarget* screening/analysis published in the subjects environmental science and chemistry in 2021 and 2022), but also by recent events such as the “International Conference on Non‐Target Screening 21” in the year 2021 in Germany. This review addressed recent and noteworthy studies concerning the processing and interpretation of NTS data acquired on LC‐HRMS instruments with a focus on the analysis of environmental samples. The landscape of tools is fairly scattered, offering plenty of specialized or partial solutions. patRoon or SLAW is a comprehensive, fully‐integrated workflow and still leave room for customization and optimization. Further efforts for making such tools more user‐friendly like GUIs, thorough documentation and quick start workflows could promote a broader use. Consistent maintenance and support even for future user generations would be of great help to the community. Comparability of results generated by different data processing tools was identified as a problem here. Future works should address this issue by using multiple feature extraction algorithms, especially when using NTS for process evaluations. Some limitations of conventional feature extraction tools are met by multivariate chemometric methods such as ROIMCR which ensures full spectral resolution when compressing second‐order LC‐MS data and does not require chromatographic deconvolution or alignment. Prioritization of relevant features is of utmost importance for NTS analysis due to the sheer amount of complex data. Strategies are mostly governed by the level of identification confidence assigned to a feature‐compound pair. Approaches that limit the number of possible annotations as was intended by the PubChemLite project could improve the efficiency of identification workflows in the future. HRMS is a powerful tool for structural elucidation by giving information on accurate mass, isotopic abundance patterns, and often fragmentation behaviour of compounds. It is probably due to these strengths that chromatographic information was sometimes overlooked in NTS identification workflows. As the RT is closely connected to the physicochemical properties of a compound like its hydrophobicity, it can be exploited for ranking or filtering annotated features. RTI and prediction make chromatographic information amenable to screening workflows. RTI tools are available on platforms like FOR‐IDENT (https://water.for‐ident.org) and the RTIs Platform of the National and Kapodistrian University of Athens (http://rti.chem.uoa.gr/). The need for additional information to augment HRMS data is also reflected by the growing interest in ion mobility spectrometry (IMS) for environmental analysis. The drift time measured by IMS is added as a fourth dimension to describe a future (besides *m/z*, RT and signal intensity) and supports the identification of chemicals of emerging concern in water samples.^77^, ^78^ Another obvious choice for prioritization of non‐target features are intensity‐based approaches. However, no direct proportionality to the analyte's concentration can be assumed due to i.e. matrix effects (signal enhancement or suppression). Normalization using ISTDs could still allow an interpretation of feature profiles along spatial or temporal axes. Finally, sophisticated predictions of ionization efficiencies are able to accurately predict a feature's concentration without the help of analytical standards. Future NTS investigations might consider shifting the focus from a primarily qualitative to a more quantitative approach which might allow a quick, provisional risk assessment of a multitude of annotated features without having to rely on reference standards. The NTS solutions became generally more applicable in our days presented in practical review articles of quantitative NTS,^71^ or other topics like IMS^79^ and GC‐MS.^80^

## CONFLICT OF INTEREST

The authors declare that they have no conflict of interest.

## AUTHOR CONTRIBUTIONS

Susanne Minkus: Conceptualization (equal), writing – original draft (lead), writing – review and editing (lead); Stefan Bieber: Conceptualization (supporting), writing – original draft (supporting), writing – review and editing (supporting); Thomas Letzel: Conceptualization (equal), writing – original draft (supporting), writing – review and editing (supporting)

## Data Availability

Data sharing is not applicable – no new data are generated.
